# Malondialdehyde in Exhaled Breath Condensate as a Marker of Oxidative Stress in Different Pulmonary Diseases

**DOI:** 10.1155/2011/891752

**Published:** 2011-06-16

**Authors:** M. L. Bartoli, F. Novelli, F. Costa, L. Malagrinò, L. Melosini, E. Bacci, S. Cianchetti, F. L. Dente, A. Di Franco, B. Vagaggini, P. L. Paggiaro

**Affiliations:** Cardiothoracic and Vascular Department, University of Pisa, Ospedale di Cisanello, Via Paradisa 2, 56124 Pisa, Italy

## Abstract

*Background*. Oxidative stress plays a role in the pathogenesis of many chronic inflammatory lung diseases. Exhaled breath condensate (EBC) collection is a noninvasive method to investigate pulmonary oxidative stress biomarkers such as malondialdehyde (MDA). *Subjects and Methods*. We measured MDA levels in EBC in a large number of patients (*N* = 194) with respiratory diseases: asthma (*N* = 64), bronchiectasis (BE, *N* = 19), chronic obstructive pulmonary disease (COPD, *N* = 73), idiopathic pulmonary fibrosis (IPF, *N* = 38). Fourteen healthy nonsmoking subjects were included as controls. *Results*. Excluding IPF subjects, MDA levels were significantly higher in all disease groups than in control group. MDA was significantly higher in COPD than asthmatic and BE subjects. Among asthmatics, corticosteroids-treated subjects had lower MDA levels than untreated subjects. COPD subjects showed an inverse correlation between MDA concentrations and FEV_1_% (rho: −0.24, *P* < .05). *Conclusions*. EBC-MDA is increased in subjects with chronic airway disorders, particularly in COPD, and it is related to FEV_1_ reduction.

## 1. Background

Oxidative stress plays an important role in the pathogenesis of many chronic inflammatory lung disorders, particularly in COPD and asthma, where it is an important consequence of irritant-induced damage of bronchial epithelial cells and represents an amplifying mechanism through the recruitment of inflammatory cells in the airways [1, 2].

Among the many biological targets of oxidative stress, membrane lipids are the most commonly involved class of biomolecules. Lipid peroxidation yields a number of secondary products able to boost oxidative damage [3]. In addition to their cytotoxic properties, lipid peroxides are increasingly recognized as being important in signal transduction for a number of events in the inflammatory response [4].

Exhaled breath condensate (EBC), obtained by cooling exhaled air, has recently been proven as a good biological tool to monitor inflammatory and oxidative stress of lower respiratory tract [5]. Several biomarkers have been measured in EBC, including aldehydes, H_2_O_2_, adenosine, isoprostanes, leukotrienes and cytokines, and an increasing number of studies report the use of EBC in the investigation of airway lining fluid composition in several respiratory diseases [6].

Malondialdehyde (MDA) has been widely studied as a product of polyunsaturated fatty acid peroxidation. High MDA levels have been observed in several biological fluids from patients with different airway diseases including asthma [7–11], COPD [11, 12], and bronchiectasis [13]. MDA in EBC has been measured in relatively few studies [9–12], and only few of them include large number of subjects [9, 10].

In this study, we measured and compared EBC levels of MDA in a large group of subjects with different pulmonary diseases in order to explore the possible usefulness of this measurement in the assessment of different lung diseases. 

## 2. Methods

### 2.1. Subjects

Exhaled breath condensate was collected from 194 subjects affected by different airway diseases attending to our pulmonary unit. Patients with asthma (A, *N* = 64), bronchiectasis (BE, *N* = 19), chronic obstructive pulmonary disease (COPD, *N* = 73) and idiopathic pulmonary fibrosis (IPF, *N* = 38) were included in the study. Fourteen healthy nonsmoking subjects were included as controls.

For each subject, diagnosis and assessment of severity for the different diseases were performed according to international guidelines recommendations [14–17]. In particular, asthmatic patients were divided according to the presence of regular treatment with inhaled corticosteroids, whereas COPD patients were divided into moderate or severe according to FEV_1_≥ or <50% predicted, respectively [15]. The diagnosis of bronchiectasis was confirmed in all patients by chest high-resolution computed tomography (HR-CT); bronchiectasis was primary or idiopathic in 13 patients, secondary in 6 patients [17]. All patients with IPF except 6 were on low-dose oral corticosteroids associated with azathioprine (in 9 patients) and/or N-acetyl-cysteine (in 23 patients). Clinical data of patients and healthy subjects are reported in Table 1.

Pulmonary function tests and other measurements used to assess diagnosis and level of severity of the different diseases were performed according to standard methods. In particular, pulmonary function tests were performed by means of a computerized spirometer (Medgraphics, Cardiorespiratory Diagnostics, St. Paul, Minn, USA) and the results were expressed as percentage of CECA (European Community for Steel and Coal) predicted values [18].

All patients were examined in a stable phase of the disease, with no acute exacerbation in the previous month. Informed consent for participation to the study was obtained from all patients. 

### 2.2. Exhaled Breath Condensate (EBC) Collection

EBC was collected cooling exhaled air with a specifically designed condenser (Ecoscreen, Jaeger, Wurzburg, Germany). Subjects breathed tidally for 15 min through a two-way non-rebreathing valve in order to prevent inspiratory and respiratory air mixing and trap saliva [5]. EBC samples were immediately stored at −80°C until analysis, which was performed within 6 months from the collection, as recommended by previous studies [9, 11]. 

### 2.3. Malondialdehyde Analysis in EBC

Malondialdehyde (MDA) concentrations were measured in EBC samples according to the method described by Lärstad et al. [19]. Samples were derivatized with thiobarbituric acid (25 mM in H_2_PO_4_ 0.3 M, pH 3.5) in a water bath at 95°C for 60 minutes, cooled in ice for 5 minutes, and then allowed to recover at room temperature for 40 minutes before analysis. 

**Figure 1:**
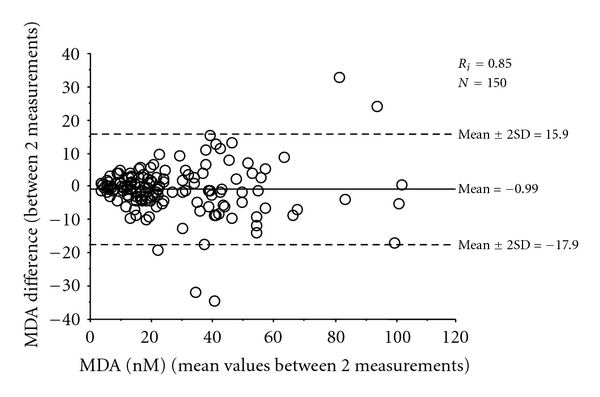
Plot of difference against mean of malondialdehyde (MDA) values obtained from two subsequent analyses. *R*_*i*_: intraclass correlation coefficient.

Analyses were performed using a high-performance liquid cromatography (HPLC) connected to a fluorescence detector (Binary HPLC pump 1525 and 2475 multi *λ* fluorescence detector, Waters, Milan, Italy), equipped with a Polaris C18 column, 150 × 4.6 mm ID, particle size 5 *μ*m (Varian, USA). Excitation and emission wavelengths were of 532 and 553 nm, respectively. The mobile phase was composed by acetonitrile: 20 mM potassium phosphate buffer pH 6.8 (25 : 75, v/v), the flow rate was 1.3 mL/min. The method had a limit of detection of 4.1 nM and a recovery of 96%. Intra- and interassay reproducibility were 1.9 and 8.7%.  Figure 1 shows the Bland-Altman scattergram of two repeated measurements of 150 samples: intraclass correlation coefficient (*R*_*i*_) was 0.854. 

### 2.4. Sputum Induction and Analysis

Sputum was performed in 108 subjects with asthma (*N* = 52), COPD (*N* = 41) and bronchiectasis (*N* = 15). Sputum induction and analysis were carried out as previously described [20, 21]. After inhaled salbutamol pretreatment, hypertonic saline solution (HS: NaCl 4.5% w/v) was delivered by means of an ultrasonic nebulizer (2.8 mL/min output; DeVilbiss Ultraneb 2000, Somerset, Pa, USA) to induce sputum production. In subjects with FEV_1_ lower than 35%, isotonic saline (IS: NaCl 0.9% w/v) was used as recommended in [20]. Nebulization was stopped after 15 min or earlier if FEV_1_ fell by 20% or more from baseline values. Before and every 5 min after the start of nebulization, FEV_1_ was measured, and patients were then asked to rinse their mouth and throat carefully to discard saliva and to try to cough sputum into a container. 

Sputum samples were processed within two hours from collection; more viscid and dense sputum portions were selected and processed as follows. Briefly, samples were homogenated by adding 0.1% (DTT) in a shaking bath at 37°C for 15 min, then centrifuged to separate cells from supernatant. The cell pellet was resuspended in PBS for viability and total cell count, and aliquots were used to prepare slides for differential cell counts. At least 300 inflammatory cells were counted. Macrophage, lymphocyte, neutrophil, and eosinophil values were expressed as percent of total inflammatory cells. Slides with cell viability <50% and an amount of squamous cells such that 300 inflammatory cells could not be counted were considered inadequate and discarded. 

### 2.5. Statistical Analysis

Functional data were expressed as mean and standard deviation and biological data as median and range. Differences between two groups were tested using unpaired *t*-test and Mann-Whitney test for normally and nonnormally distributed variables, respectively. Differences among three or more groups were tested using ANOVA and Kruskal-Wallis test for normally and nonnormally distributed variables, respectively. Correlations between functional and biological indices were evaluated using Spearman's rank correlation test. A *P* value lower than  .05 was considered as significant.

Intraclass correlation coefficient was calculated to assess MDA interassay reproducibility (*R*_*i*_ values over 0.70 are usually interpreted as satisfactory) and the Bland-Altman plot was used to graphically represent the variability between the two measurements [22]. The limits of agreement were expressed as ±2 standard deviations (SDs) of the mean of differences between the two measurements within which 95% of the repeated measures are expected to lie. 

## 3. Results

**Figure 2:**
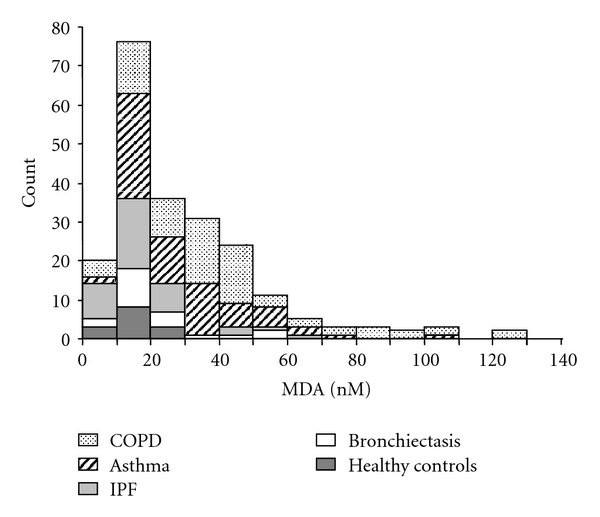
Distribution of Malondialdehyde (MDA) in the different categories of subjects. COPD: chronic obstructive pulmonary disease; IPF: Idiopathic pulmonary fibrosis.

The distribution of all measurements of MDA in EBC in normal subjects and in patients with different diseases is reported in Figure 2. Healthy subjects and patients with IPF were distributed in the lower range of MDA, while COPD patients were mainly represented in the categories with higher MDA concentrations.

**Figure 3:**
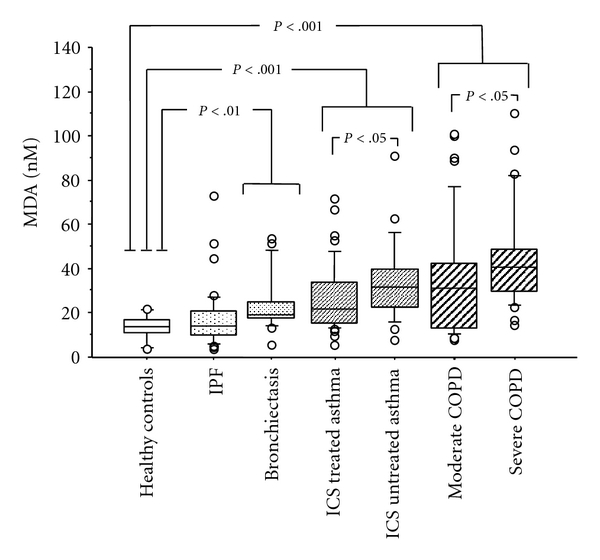
Median values (interquartile range) of malondialdehyde (MDA) in EBC in the different categories of patients with different pulmonary diseases and healthy subjects; IPF: Idiopathic pulmonary fibrosis; ICS: inhaled corticosteroids; COPD: chronic obstructive pulmonary disease.

Except for IPF patients, who showed MDA levels similar to those of healthy controls (15.2 (4–79) versus 15.6 (4–25) nM, resp.), all the disease groups showed higher levels of MDA than healthy controls (Figure 3). Among asthmatics, no difference in MDA was observed between difficult-to-control and mild/moderate asthma (26.0 (14–72) versus 26.1 (6–91) nM). Comparing asthmatic patients according to treatment, we found that ICS-treated subjects had lower levels of MDA than untreated subjects (21.5 (6–72) versus 32.0 (8–91) nM, *P* < .05). COPD patients had significantly higher levels of MDA than asthmatic and BE subjects (37.0 (8–126) versus 26.3 (6–91) nM, *P* < .05, and 19.2 (6–54) nM, *P* < .05, resp.). Within the COPD group, subjects with severe COPD showed higher levels of MDA than patients with moderate COPD (39.0 (8–126) versus 29.1 (9–81) nM, resp., *P* < .05).

**Figure 4:**
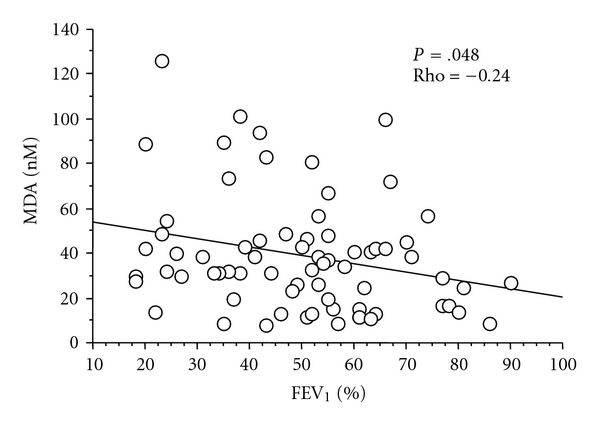
Correlation between FEV_1_, % predicted, and malondialdehyde (MDA) concentrations in COPD subjects (Spearman rank correlation test).

An inverse correlation between MDA concentrations and FEV_1_ was observed both in the whole group of patients (*P* < .001, rho: −0.25) and in the COPD subgroup (*P* < .05; rho: −0.24) (Figure 4). No difference in MDA was found dividing the whole group of patients according to their smoking status (*P* = .52). Considering each disease group separately, we found that among COPD patients, MDA levels in current smokers were not different from ex- and nonsmokers (*P* = .11),   whereas among asthmatics, current smokers had higher MDA levels than ex- and nonsmokers (38.5 (19–91) versus 25.5 (6–72) nM, *P* < .05).

**Figure 5:**
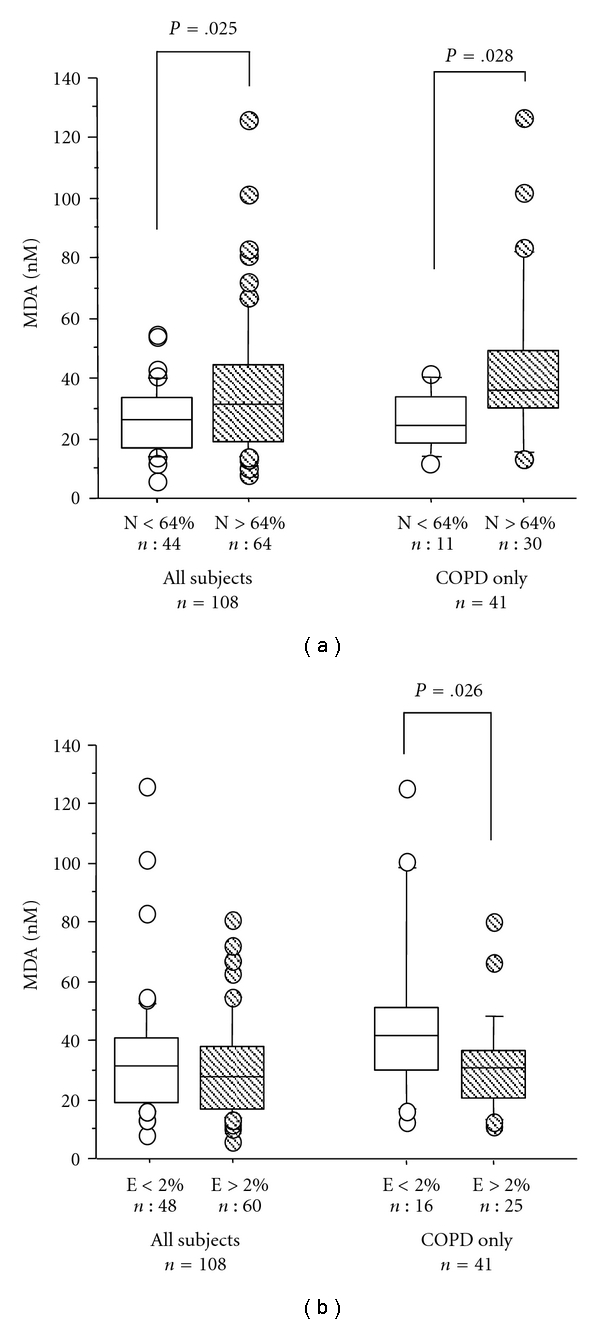
Median values (interquartile range) of malondialdehyde (MDA) in EBC according to induced sputum percentage of neutrophils (N%) (a) and eosinophils (E%) (b) in all patients and in COPD patients only. COPD: chronic obstructive pulmonary disease.

In the subgroup of 108 patients who underwent induced sputum analysis, no relationship between EBC levels of MDA and sputum inflammatory cell counts was found. However, patients with high (>64% [23]) sputum neutrophils showed significantly higher MDA levels (*P* < .05) than patients with low sputum neutrophils, even when COPD patients only were considered (Figure 5(a)). No difference for MDA values was found between subjects with high (>2% [23]) or low sputum eosinophils in the whole group of patients, but COPD subjects with high sputum eosinophils had lower MDA levels (Figure 5(b)). 

## 4. Discussion

We found that subjects with chronic airway disorders have increased levels of MDA in EBC, thus confirming that oxidative stress plays a role in the pathophysiology of lung diseases. We also found that MDA concentrations in EBC are related to FEV_1_ and neutrophilic inflammation, particularly in COPD patients. 

MDA is a marker of oxidative stress that can be measured, using different and sometimes complex analytical procedures [10, 11], in blood, sputum, bronchoalveolar lavage fluid, and EBC. Lipid mediators, in fact, at body temperature gain some volatility, thus becoming detectable also in EBC. Some authors have shown a relationship between MDA and other markers of severity of airway diseases [24, 25] as well as its sensitivity to the effect of treatment [26, 27], but these data have not been confirmed by others [11]. This discrepancy may be ascribed to the different populations studied (considering also the heterogeneity of asthma and COPD) and to the different methods of measurements used. 

In our experience, MDA measurement in EBC was reproducible and accurate: interassay reproducibility was good and not different from what reported by other authors using different, more complex methods [11]. We checked storage stability of EBC samples, just in few samples showing a good repeatability; however, we analyzed all samples within 6 months from the collection, as suggested by the international recommendations and previous papers [5, 9, 11].

Our method differentiates normal subjects from patients with different airway diseases despite some overlapping of single measurements. Furthermore, MDA was sensitive to changes induced by therapeutic intervention, as shown by the lower levels of MDA in asthmatics treated with ICS in comparison with untreated asthmatics. This issue is controversial in literature: while some studies reported a reduction in MDA levels after corticosteroid treatment [24], other studies found no difference between ICS-treated and untreated subjects [11]. Antioxidant treatment has been generally reported to be ineffective in modifying markers of oxidative stress [28, 29]. In a preliminary study, we observed a mild, significant attenuation in ozone-induced EBC-MDA increase after 4-week treatment with oral N-acetyl-cysteine in asthmatic patients [30]. 

COPD subjects showed the highest values of MDA in EBC. This observation was expected, considering that oxidative stress plays a relevant role in smoke-induced airway damage and chronic inflammation. This was confirmed by the higher levels of MDA in patients with higher percentage of sputum neutrophils, and by the inverse correlation between EBC-MDA and FEV_1_. These results are in agreement with other reports [11], suggesting that MDA may be a good marker of the severity of the disease. Similar results were obtained in COPD when other markers of oxidative stress were considered (such as 8-isoprostane, hydrogen peroxide, LTB4, or other COPD-related cytokines) [31–33]. No difference was observed between current smoker and ex- or nonsmoker COPD patients, and this might be due both to the small number of current smokers and to the similar pattern and degree of airway inflammation in COPD patients regardless of their smoking habit [34].

In asthmatic patients, EBC-MDA levels were lower than those observed in COPD patients, but higher than normal subjects. There was no difference according to asthma severity, but ICS treatment was associated with lower values of MDA. While some authors demonstrated a significant decrease in blood MDA during the recovery from asthma exacerbation treated with oral corticosteroids [24], other authors did not find any difference between ICS-treated and untreated asthmatic patients [11]. In our asthmatics, current smokers showed higher levels of EBC-MDA than nonsmokers; this observation is in agreement with the prominent neutrophilic airway inflammation in smoking asthmatics [35].

In bronchiectasis, we found EBC-MDA levels slightly higher than normal subjects, but lower than COPD patients. This result may be unexpected, considering that sputum neutrophilia is a prominent feature of this kind of patients. We found no papers evaluating EBC-MDA levels in bronchiectasis, and only one paper reports increased blood MDA levels in children with bronchiectasis [13]. Increased levels of other markers of oxidative stress (such as hydrogen peroxide or pH) in EBC have been reported for bronchiectasis [36, 37].

In IPF, no difference in EBC-MDA levels was observed in comparison with healthy subjects. In the literature, few data are reported showing EBC increase of hydrogen peroxide, 8-isoprostane and nitrates in comparison with healthy subjects [38, 39]. The low levels of EBC-MDA observed in our study may be explained by the fact that most IPF patients were on oral corticosteroid and/or N-acetyl-cysteine treatment. 

## 5. Conclusions

In conclusion, we demonstrated that MDA may be accurately measured in EBC of most patients with chronic pulmonary diseases and that it is partially related to the severity of airway obstruction and neutrophilic inflammation. This was particularly evident in COPD patients, where oxidative stress seems to play a major role in the pathophysiology of the disease. Thus, MDA may be a promising marker of oxidative stress in asthma and COPD. Its usefulness in the management of these diseases, as well as for other inflammatory markers, needs to be explored with well-designed, specific studies. 

## Figures and Tables

**Table 1:** Main characteristics of the groups under study.

Disease	No	Age	Gender (M/F)	Smoking habit (yes/no/ex)	FEV_1_ (% pred)	ICS therapy (yes/no)
Healthy controls	14	40.4 ± 13.1	6/8	0/14/0	102.4 ± 8.9	0/14
Asthma (all)	64	45.8 ± 16.5	28/36	7/45/12	92.6 ± 13.9	41/23
ICS untreated	23	42.0 ± 14.7	8/15	3/14/6	99.0 ± 10.7	0/23
ICS treated	41	47.2 ± 17.7	20/21	4/31/6	88.7 ± 14.2	41/0
COPD (all)	73	71.1 ± 7.6	60/13	18/9/46	49.8 ± 17.7	73/0
Moderate	38	71.6 ± 7.3	29/9	11/6/21	63.3 ± 10.6	38/0
Severe	35	69.8 ± 7.8	31/4	7/3/25	34.3 ± 9.9	35/0
IPF	38	70.2 ± 7.4	16/22	3/19/16	78.0 ± 21.2	9/29
Bronchiectasis	19	55.5 ± 13.0	5/14	0/14/5	87.3 ± 22.0	13/6

FEV_1_: forced expiratory volume 1 sec; ICS: inhaled corticosteroids; COPD: chronic obstructive pulmonary disease; IPF: idiopathic pulmonary fibrosis.

## Abbreviations

EBC: Exhaled breath condensate
MDA: Malondialdehyde
HPLC: High-performance liquid cromatography
COPD: Chronic obstructive pulmonary disease
IPF: Idiopathic pulmonary fibrosis
BE: Bronchiectasis
FEV_1_: Forced expiratory volume in one second
H_2_O_2_: Hydrogen peroxide
CECA: European Community for Steel and Coal
*R*_*i*_: Intraclass correlation coefficient
HS: Hypertonic saline
IS: Isotonic saline
DTT: Dithiothreitol
PBS: Phosphate buffer solution
ICS: Inhaled corticosteroids
N: Neutrophils
E: Eosinophils
LTB4: Leukotriene B4.
